# Thoracic electrical impedance tomography identifies heterogeneity in lungs associated with respiratory disease in cattle. A pilot study

**DOI:** 10.3389/fvets.2023.1275013

**Published:** 2024-01-04

**Authors:** Olivia Brabant, Yuliya V. Karpievitch, Alphons Gwatimba, William Ditcham, Ho Yin Ho, Anthea Raisis, Martina Mosing

**Affiliations:** ^1^School of Veterinary Medicine, Murdoch University, Perth, WA, Australia; ^2^Wal-yan Respiratory Research Centre, Telethon Kids Institute, Perth, WA, Australia; ^3^School of Biomedical Sciences, University of Western Australia, Perth, WA, Australia; ^4^Anesthesiology and Perioperative Intensive Care, Department for Companion Animals and Horses, University of Veterinary Medicine Vienna, Vienna, Austria

**Keywords:** electrical impedance tomography, cattle, imaging, respiratory disease, thorax

## Abstract

Respiratory disease in cattle is a significant global concern, yet current diagnostic methods are limited, and there is a lack of crush-side tests for detecting active disease. To address this gap, we propose utilizing electrical impedance tomography (EIT), a non-invasive imaging technique that provides real-time visualization of lung ventilation dynamics. The study included adult cattle from farms in Western Australia. The cattle were restrained in a crush. A standardized respiratory scoring system, which combined visual, auscultation, and clinical scores, was conducted by two non-conferring clinicians for each animal. The scores were blinded and averaged. During assessment, an EIT electrode belt was placed around the thorax. EIT recordings of ten suitable breaths were taken for analysis before the cattle were released back to the herd. Based on the combined examination scoring, the cattle were categorized as having healthy or diseased lungs. To allow visual interpretation of each breath and enable the creation of the quartile ventilation ratio (V_QR_), Flow/Tidal Impedance Variation curves (F/TIV) were generated for each breath. The analysis focused on two EIT variables: The novel V_QR_ over time during inhalation and exhalation and global expiratory impedance (TIV_EXP_) adjusted by breath length. A mixed effects model was used to compare these variables between healthy and diseased cattle. Ten adult cattle of mixed ages were used in the current analysis. Five cattle were scored as healthy and five as diseased. There was a significant difference in the examination scores between the healthy and diseased group (*P* = 0.03). A significant difference in V_QR_ during inhalation (*P* = 0.03) was observed between the healthy and diseased groups. No difference was seen in V_QR_ over time during exhalation (*P* = 0.3). The TIV_EXP_ was not different between groups (*P* = 0.36). In this study, EIT was able to detect differences in inhalation mechanics when comparing healthy and diseased cattle as defined via clinical examination, highlighting the clinical utility of EIT.

## 1 Introduction

Respiratory disease in cattle is of global importance, with significant production and financial implications. In Australia, the significant economic impact equates to losses in the feedlot industry of $40 AUS million annually (1). Bovine respiratory disease syndrome (BRDS) has a multifactorial pathophysiology combining host, environment, and agent interactions. This syndrome impacts both the beef and dairy industry, affecting up to 10% of immature cattle and 2.5% adult cattle (2). Bovine respiratory disease syndrome is responsible for 60–90% of morbidity and mortality in feedlots and is the most common disease syndrome in cattle (3–6).

Current diagnostic methods for BRDS are predominantly limited to a thorough history and clinical examination. However, studies have shown that 35–50% of cattle exhibiting lesions in the lungs at slaughter had no history of disease and 64% with lesions at slaughter had received no treatment for BRDS (7–9). Methods to try and improve clinical diagnosis have been developed to include scoring scales such as the Wisconsin calf scoring scale and biomarkers for disease (10–12). However, with these methods specificity and sensitivity remains low (6, 13).

Imaging techniques for identifying lung disease is mainly limited to ultrasound, but in small calves there is the potential to use radiography and computed tomography (CT) (14–16). CT is not possible in adult cattle due to issues of the size of the gantry and is cost prohibitive in young and adult cattle (17–19). Although ultrasound can be utilized in both young and adult cattle, identification of lesions is limited to the pleural surface and superficial lung parenchyma, leaving deeper lesions undiagnosed (18).

More recently there have been developments in alternative monitoring tools to investigate lung ventilation in calves and adult cattle using electrical impedance tomography (EIT) (20, 21). These studies have shown that EIT can monitor ventilation in cattle, although assessment of changes in regional ventilation in the presence of respiratory disease has not been performed.

EIT is a non-invasive imaging modality that uses imperceptible alternating currents to detect impedance changes where there is an alteration in electrical conductivity in the body. Such alterations are due to changes in regional distribution of ventilation within the lung, or changes in blood flow (22, 23). These impedance changes can be reconstructed into real-time images of ventilation distribution, and perfusion, through the presence of gas or blood and tissue stretch, influences those impedance changes (22–24). Numerous studies support its use for investigating lung pathology, such as pneumothorax in humans (25), pathologic ventilatory incidents created in porcine models (26), and acute respiratory distress syndrome (27, 28), including that associated with the recent COVID-19 outbreak (29, 30).

Thus far, there have been studies in veterinary medicine to explore ventilation and perfusion dynamics in healthy conscious and anesthetized animals (20, 31–34). Respiratory studies in horses have used the steepness of the flow/tidal impedance variation curve (F/TIV) and intercept on the expiratory F/TIV curve before and after histamine provocation and salbutamol nebulization to explain differences in the shape of the curve. However, there has only been one study investigating pathology in horses suffering from left-sided cardiac volume overload (35) and no investigation to date of lung pathology in cattle.

The aim of this paper is to evaluate if EIT variables can detect a difference in the homogeneity of ventilation between healthy cattle and cattle with respiratory disease. Our hypothesis is that, by employing EIT, we will assess respiratory mechanics in cattle that can be associated with disease develop EIT-based classification metrics that will correlate with standard clinical examinations and provide additional lung ventilation information.

## 2 Methods

### 2.1 Ethics statement

This prospective experimental study was performed with the approval of the Murdoch University Animal Ethics Committee (R3275/20). The Australian code for the care and use of animals for scientific purposes was followed.

### 2.2 Animals

Data were collected from two dairies and three beef suckler units in the Perth metropolitan and Southwest regions of Western Australia. The farms were contacted prior to the study; they were a mixture of veterinary teaching farms, clients of the university practice, and external contacts. They were provided with an overview of the study and proposed outcomes and agreed to participate prior to commencement of the study. The farms were selected to cover diversity in conformation using a mixture of beef and dairy cattle. Suitability of participation was assessed prior to commencement of the study to ensure all animals were used to being handled and spending time restrained in a crush.

The participating farms sourced animals with and without suspected respiratory disease based on their observations. Five healthy animals (no previous treatment for respiratory disease) and five animals exhibiting clinical signs of respiratory disease were selected to participate in the initial pilot study from a larger cohort of 83 animals. The cattle were assigned a letter from A through to J and categorized into the diseased or healthy group post examination, prior to the analysis. Prior to the study, the animals were on pasture and subject to routine management on the prospective farms before being brought to the handling facilities. Each animal was restrained in a standing position within a crush and hosed down with water before EIT electrode belt placement. All animals tolerated belt placement well to allow reliable data to be collected.

### 2.3 Instrumentation and data collection

**Figure 1:**
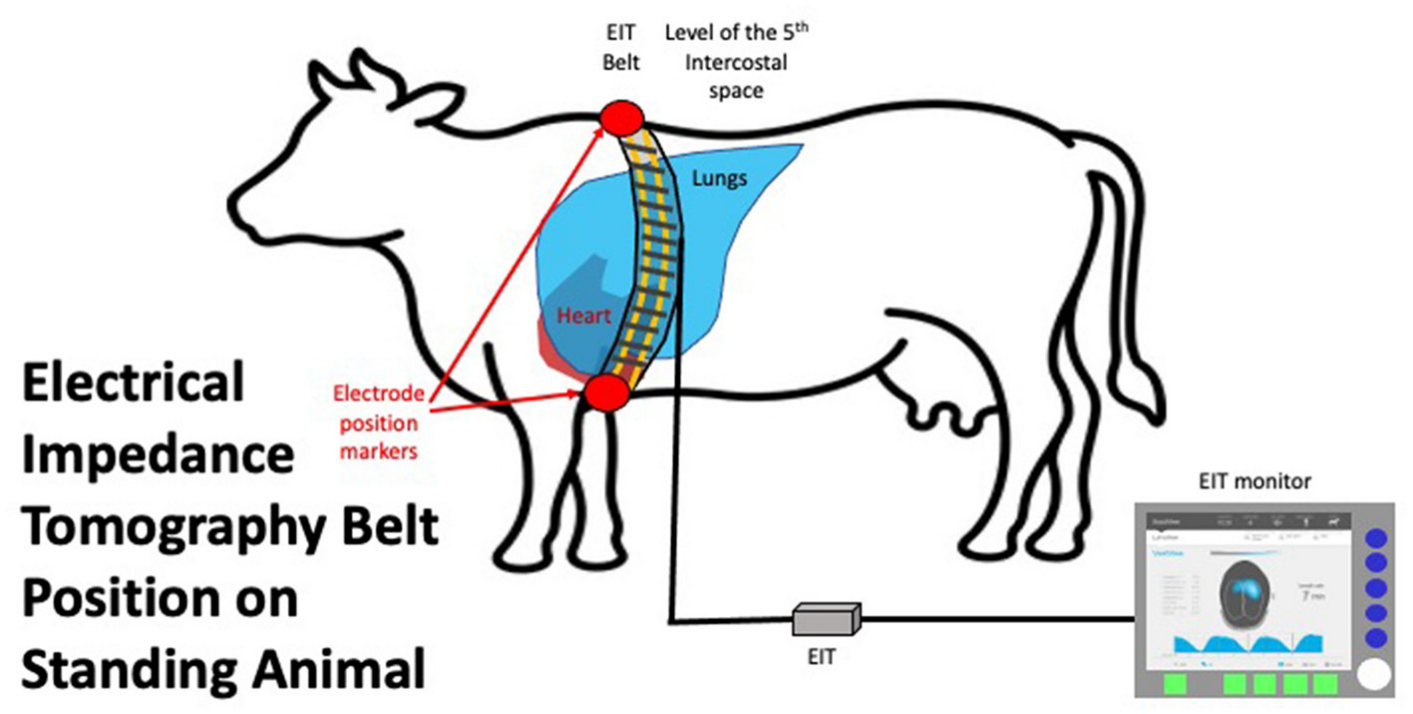
EIT belt *in situ* on an animal in standing position placed vertically at the level of the 5th intercostal space. The connector to the EIT control unit is shown on the **left** of the image. The EIT control unit is connected to a laptop with data recording and basic analysis software.

The stretchable EIT belt, with 32 electrodes mounted equidistantly, was prepared by placing low-conductive ultrasound gel (Aquasonic^®^, Fairfield, USA) onto each electrode prior to placement on the animal. The belt was placed circumferentially on the animal at the level of the 5th intercostal space (ICS) and fastened on the ventral aspect. Care was taken to ensure the belt was vertical and the middle of the belt between electrode 16 and 17 was at the point of the withers. The belt was connected to the EIT device (Sentec, Landquart, Switzerland), which was connected to a laptop to allow data recording using BBVet Software (Sentec, Landquart, Switzerland) (Figure 1). All cattle were subject to at least 2 min of measurements to allow 10 consecutive breaths to be collected without movement artifacts or failing electrodes. After completion, the recording was saved to be analyzed retrospectively. The belt was removed and the animal was released from the crush and returned to the herd.

### 2.4 Data analysis

#### 2.4.1 Clinical examination analysis

The circumference, weight, and body condition score (thin-obese: 1–5 using quarter scores) of each animal was measured prior to placement of the belt. Respiratory clinical examinations were performed in accordance with a modified Wisconsin calf scoring scale modified for adult cattle by Blakebrough-Hall et al. (10). Two experienced veterinarians who were blinded to healthy or diseased status, and each other, completed the examinations. Separate recording sheets were used with no conferring. The order in which each veterinarian examined each animal was randomized. A number was drawn out of a blinded envelope for each animal, stating either one or two to indicate which veterinarian should examine the animal first. A clinical examination was conducted in three parts. The visual clinical signs (Part A), including head carriage, labored breathing, nasal discharge, ocular discharge, rumen fill, and lethargy, were allocated a score of 0–3 (unremarkable-profound changes for each parameter). A diseased animal was classified as having an averaged visual score ≥3.5 out of a possible total of 25. A separate lung auscultation (Part B) scored 0–3 (no abnormalities-abnormal lung sounds throughout the entire lung fields and prolonged respiratory sounds). A diseased animal was classified as having a lung auscultation ≥1 from an average of both examiners. The final part of the examination was the clinical score (Part C), where rectal temperature (0 <40–1 ≥ 40°C) and respiratory rate (0 <40 breaths per minute-1 > 40 breaths per minute) were scored. A diseased animal was classified as having a clinical score ≥1 with a maximum of two. The cut off for a diseased animal was a visual score ≥3.5, a lung auscultation score of ≥1, and a clinical score ≥1. Scores from both examiners were averaged to give a visual and clinical diagnosis and categorized into healthy or diseased.

#### 2.4.2 EIT analysis

ElT recordings were taken from all cattle and retrospectively analyzed using reconstruction software (IBEX, Sentec Landquart, Switzerland) to find 10 representative breaths. At least three representative, movement artifact-free breaths from each animal were used for further analysis (36). The EIT analysis was initially performed with the researcher blinded to the results of the clinical examination to eliminate sampling bias of breath selection.

The EIT data from ten cattle were further analyzed using a custom script [written in Python 3.10.8, (CreateSpace, Scotts Valley, California)] and visualized using R 4.2.2 (Development Core Team 2020, GNU, Public license, USA) with all pixels within the image used in the analyses. First, the EIT files for all cattle were opened in IBEX and the impedance data corresponding to the appropriate breaths (up to 10 breaths per EIT file) were exported as .MAT files for external processing. These .MAT files were then processed using Python scripts using packages SciPy (37), NumPy (38), Pandas (39), and Matplotlib (40). Specifically, the impedance and rate of change of impedance across time for each breath was computed to generate Flow/Tidal impedance variation (F/TIV) curves which were exported as .CSV files for statistical analysis in R.

#### 2.4.3 EIT variables

1) Flow/Tidal impedance variation curve (F/TIV)

**Figure 2:**
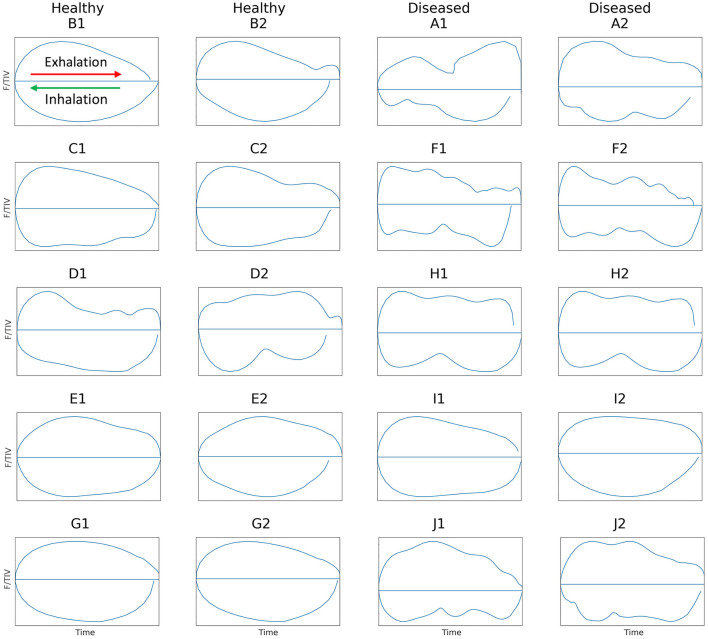
Flow/tidal impedance variation (F/TIV) curves in ten cattle illustrating two breaths from each animal. Inhalation is shown in the **bottom** curve of each breath moving **right to left** (green arrow) and exhalation is shown in the **top** half of each breath moving **left to right** (red arrow). Healthy cattle are shown in the first two columns on the **left** with smooth F/TIV curves and diseased cattle are in the last two columns to the **right** with irregular curves illustrating multiple peaks in each breath.

F/TIV describes the impedance and rate of change in impedance across time in a single breath. The global TIV is given, rather than volume, as no calibration of impedance change to a corresponding change in air to volume has been determined in cattle. Linear relationships between change in impedance and change in the volume of air in the lungs have been seen (31, 41). It is assumed that impedance change correlates to changes in volumes of gas in the lung regions over a single breath (42). To generate a flow parameter, we have used impedance change as a surrogate for volume, to allow the first derivative of which to be analogous to flow (F). The curve is generated by plotting the average impedance value of all pixels in each 2-D EIT frame during the entire breath (TIV) against the 1st derivate of the TIV, flow (F) over time, measured in arbitrary units, as no calibration to volume has been conducted. The shape of this curve was used to identify differences between lung disease states. Scaling was employed where the total impedance change was smaller, to allow visualization and description of the curve. A smooth semicircular curve with few or no peaks/troughs in inhalation and exhalation was observed in healthy animals and irregular-shaped curves that showed multiple peaks in both inhalation and exhalation were noted in diseased cattle (Figure 2).

2) Global expiratory impedance variation (TIV_EXP_)

TIV_EXP_ describes the impedance change of all pixels between the beginning and end of expiration of all pixels within the lung region of interest (ROI). The TIV_EXP_ is calculated for each breath by subtracting the impedance at the beginning of exhalation from the impedance at the end of exhalation in all pixels, is normalized to breath length, and is displayed in arbitrary units.

3) Quartile ventilation ratio (V_QR_ )

**Figure 3:**
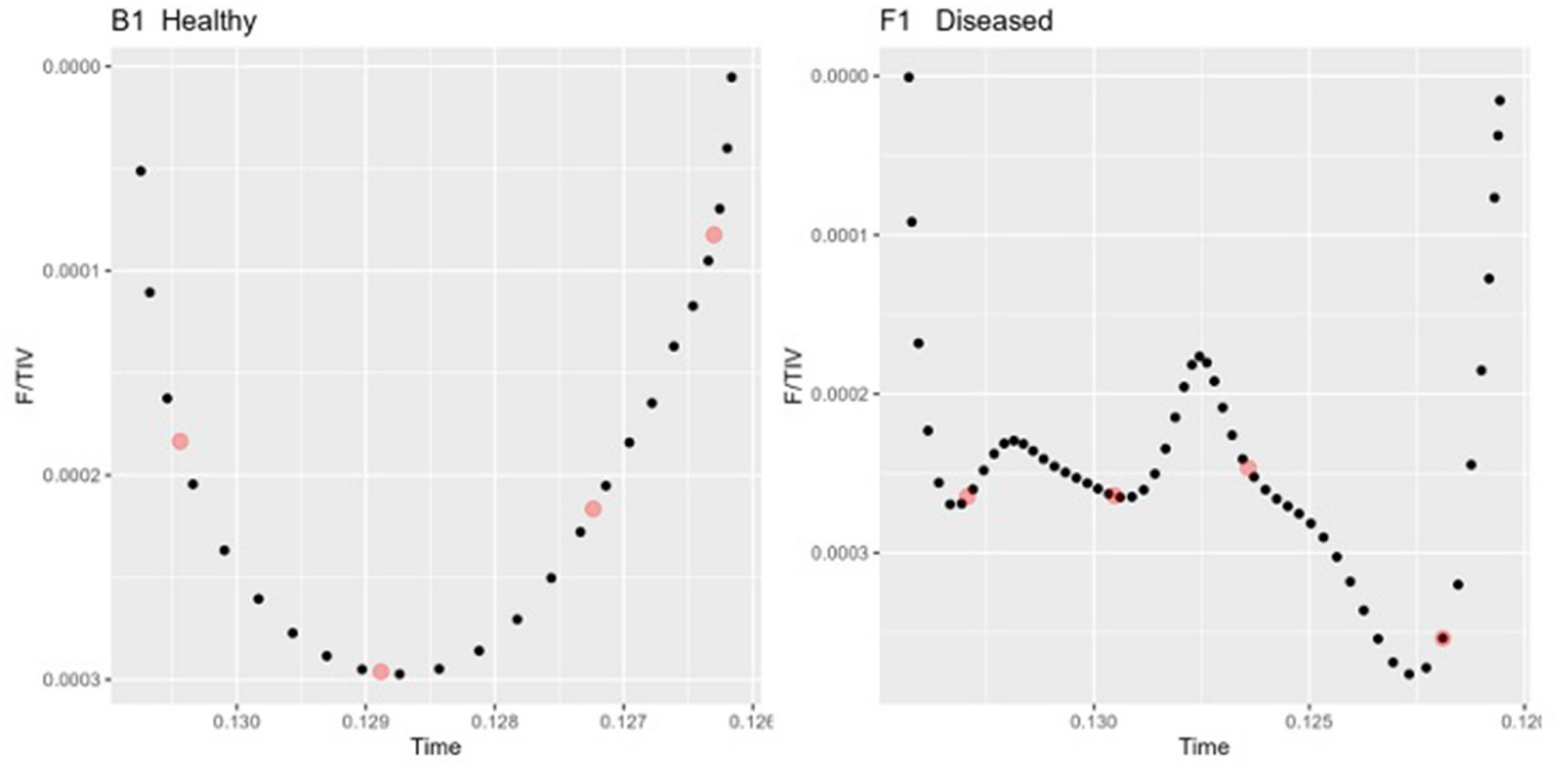
Two inhalation sections of the Tidal impedance variation (TIV) curves over time in one healthy **(left)** and one diseased animal **(right)**. The dots (red) indicate the points taken as the medians of each quartile Q1-Q4 used to compute the Quartile Ventilation Ratio (V_QR_).

To describe the changes in the shape of the F/TIV curves, we developed a novel numerical measure, V_QR_, to describe the inhomogeneity in lung filling over time. V_QR_ is the ratio of total impedance change between the first 25% and last 25% of inhalation or exhalation with the middle 50% of inhalation or exhalation: The F/TIV curves were divided into quartiles (0–25% = Q1, 25–50% = Q2, 50–75% = Q3, 75–100% = Q4) and the median point (Figure 3) on the F/TIV curve of each quartile was used to create a ratio of the sum of Q1 and Q4 and the sum of Q2 and Q3. The V_QR_ was measured for all breaths for that individual to create a fractional rate of change (Equation 1) (Figure 3). The V_QR_ was calculated for inhalation V_QRi_ and exhalation V_QRe_.


$$\begin{array}{l} V_{QR}=\frac{\left(Q1+Q4\right)}{\left(Q2+Q3\right)} \end{array}$$

### 2.5 Statistical analysis

Statistical analysis of the veterinary scores were performed using R statistical software version 4.2.2 (Development Core Team 2020, GNU, Public license, USA). Descriptive statistics (median IQR) of visual scores, lung auscultation scores, temperature, respiratory rate, weight, circumference, and body condition scores are shown in Table 1. Agreement between the two scorers was conducted. Non-parametric tests were used to analyse the data that was not normally distributed. Wilcoxon Rank Sum Test and multiple testing adjustment using Banjamini-Hochberg adjustment were used to describe the differences between diseased and healthy visual scoring, lung auscultation, and clinical score (Table 1). The EIT data was analyzed using a linear mixed effects model to explore differences between the diseased and healthy groups, using R statistical software and the ImerTest package (43). Wilcoxon Rank Sum Test was used for the V_QRe_ and V_QRi_ statistical analysis after averaging V_QR_ for each breath within each animal. A *p*-value of <0.05 was considered significant.

**Table 1:** Median and Interquartile range (IQR) of the ten analyzed cattle, showing the visual and respiratory scores, temperature, respiratory rate, weight, circumference of the thorax, and body condition score of healthy and diseased cattle.

**Cows analyzed *N* = 10**	**Visual score (0–21)**	**Lung auscultation (0–3)**	**Clinical score (0–2)**	**Temperature (°C)**	**Respiratory rate (BPM)**	**Weight (Kg)**	**Circumference of thorax (cm)**	**Body condition score (1–5)**
Healthy (*n* = 5) (IQR)	2 (0.5)^*^	0 (0)^*^	0 (0)^*^	38.9 (0.5)	32 (0)	610 (72)	210 (15)	2.75 (0.25)
Diseased (*n* = 5) (IQR)	6 (1)^*^	1.5 (0.5)^*^	1 (0)^*^	39.1 (0.7)	46 (4)	471 (151)	189 (22)	2.5 (1)
*P*-values	(*p*-0.03)	(*p*-0.03)	(*p*-0.03)	(*p*-0.4)	(*p*-0.05)	(*p*-0.06)	(*p*-0.06)	(*p*-0.06)

^*^p < 0.05.

## 3 Results

Ten cattle were examined, respiratory disease assessment was conducted, EIT images taken, and results displayed as median (IQR). Nine adult female cows and one male steer of mixed ages [2.5 (0.55) years], with a weight of [552 (430) kg] and body condition score of [1.75 (2)], were included in the study. Of the ten animals, five were classified as diseased, namely cattle B, C, D, E, and G, and five cattle were classified as healthy, namely cattle A, F, H, I, and J. Diseased cattle had a visual score equal to or more than 3.5, a lung auscultation equal to or more than 1, and a clinical score more than 1. The maximum total score including visual score lung auscultation and clinical score in the healthy animals was 2.5 with a variance (s^2^ = 0.14) and the maximum in the diseased animals was 15.5 (s^2^ = 15.88) out of a possible 25. Agreement between the two scorers was conducted with correlation of scores for visual score (*R* = 0.93) and lung auscultation (*R* = 0.82), respectively.

An overall difference was observed between the healthy and diseased cattle (*p* = 0.03). Differences were observed from visual score (*p* = 0.03), lung auscultation (*p* = 0.03), and clinical score (*p* = 0.03), respectively. No difference was seen for weight, thorax circumference, or body condition score between healthy and diseased cattle (Table 1).

**Figure 4:**
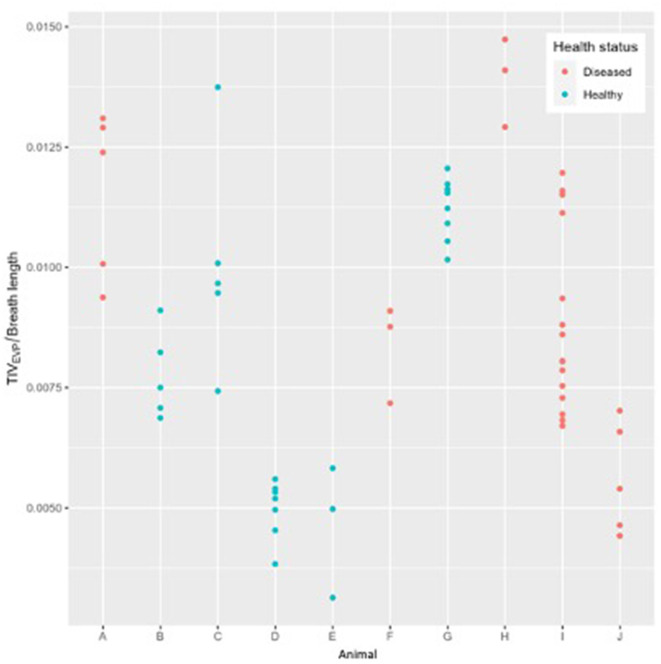
Expiratory tidal impedance variation (TIV_EXP_) by breath length (arbitrary units) for five healthy cattle (green) and five diseased cattle (red) examining up to ten breaths from each animal.

**Figure 5:**
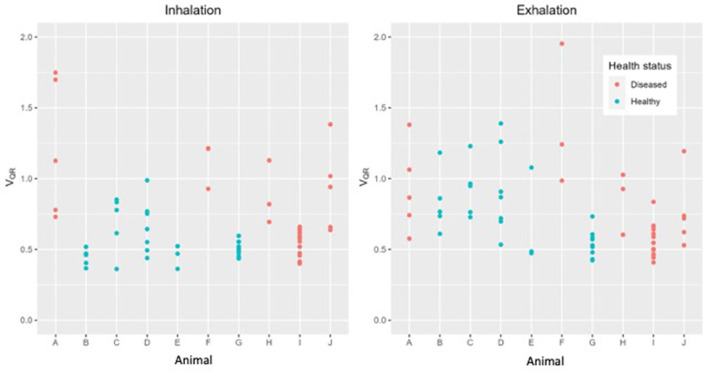
Quartile ventilation ratio (V_QR_) in ten cattle during inhalation **(left)** and exhalation **(right)**. During inhalation V_QR_ differentiates between healthy (green) and diseased (red) cattle. Each dot represents a single breath, up to ten breaths have been analyzed in each animal.

There was no statistical difference in TIV_EXP_ between healthy and diseased cattle (*p* = 0.36) (Figure 4). Exhalation V_QRe_ (*p* = 0.3) also did not show a difference between healthy and diseased cattle (Figure 5; Table 2). By comparison, inhalation V_QRi_ (*p* = 0.03) did show a difference between healthy and diseased cattle (Figure 5; Table 2).

**Table 2:** Results of the mixed-effects model comparison of diseased vs. healthy cattle using the quartile ventilation ratio (V_QR_) during inhalation and exhalation.

**Cows analyzed *N* = 10**	**Estimate**	**Std. error**	**Degrees of freedom (df)**	***t*-value**	**Pr (>|t|)**
V_QRi_ (*n* = 10) inhalation	−0.351	0.128	8.421	−2.755	0.03^*^
V_QRe_ (*n* = 10) exhalation	−0.136	0.181	7.76	−0.755	0.3

^*^p < 0.05.

## 4 Discussion

In this study, we utilized existing knowledge of lung pathology and physiology in cattle to investigate alterations in the homogeneity of ventilation using EIT that may be associated with respiratory disease. We showed that expiratory tidal impedance variation adjusted to breath length illustrated no difference between healthy and diseased populations. However, inhomogeneous ventilation and filling was detected between the two populations of cattle when comparing quartile ventilation ratio (V_QRi_) during inhalation. Thus, our study is the first to report the ability of EIT to detect differences between healthy and animals known to be diseased, when compared to the results from standardized clinical examination of cattle.

Due to both the unique anatomy cattle possess compared with other species, and the fact that the majority of lung disease occurs as a result of inhaled pathogens, it was expected that we would observe inhomogeneous lung ventilation and filling rates in the diseased cattle. Our results reflect this inhomogeneity in filling over time. Anatomically, cattle lungs contain separate lung lobules with well-developed interlobular septa, preventing pathogens from passing easily from one lobe to the next (44). Cattle, unlike small animals such as dogs, have reduced collateral ventilation (45, 46). This reduces the ability of the lungs to shift ventilation to other lobes, especially when pathology is present (47–49), leading to inhomogeneous filling.

Respiratory disease of the lung tissue causes a decrease in the ability of alveoli to open, requiring elevated opening pressures to fill (50). This can also change the time constants of lung units, where one lung unit is opening more slowly than others when pathology is present. This is especially true in cattle due to their unique anatomy of the lung, as described above. Based on these physiologic facts, it is not surprising that a significant difference was found between healthy and diseased cattle for a variable describing the homogeneity of lung filling rate. Conversely, expiration is considered passive, and therefore disease of the lung tissue is less likely to lead to variation in emptying times (51). In this study no difference in the expiratory EIT variables was found.

We observed significant differences between healthy and diseased cattle populations when comparing clinical respiratory disease scoring to novel algorithms detecting the inhomogeneity of filling using EIT.

In one animal (D) deemed healthy, the inhalation curve had more troughs than the other four healthy cattle, suggesting that this animal may have been diseased. On the other hand, for an animal (I) considered diseased, the inhalation and exhalation of both breaths F/TIV curves were smooth, suggesting that this animal was healthy (Figure 2). This suggests that F/TIV curves may be more sensitive than the clinical examination, or not as representative in cattle as in horses (42). Reviewing the clinical examination, there was no evidence to suggest that animal D was unhealthy, however, the V_QR_ suggested this animal did show evidence of respiratory disease. The results of the examination of data for animal I data confirmed significant nasal discharge and a low head carriage with labored breathing and a positive auscultation and clinical score, yet the V_QRi_ and V_QRe_ were more representative of a healthy animal or an animal with focused upper respiratory tract disease. Clinical scoring has limited sensitivity, as explained in the introduction (7, 9) however, one explanation for the lower V_QR_ could be disease contained within the upper respiratory tract, such as chronic granular rhinitis or Infectious Bovine Rhinotracheitis (IBR), that predominantly affects the upper airways (52, 53). If these conditions were chronic or recrudesced as part of a carrier status, then the animal in question may have compensated for this and was able to inhale effectively despite the increase in resistance to the airways. Another reason could be environmental: heavy dust loads during summer in West Australia may cause nasal discharge, lowered head carriage, and labored breathing yet there may be no underlying lung pathology. Analysis of further animals will allow further explanation of these outliers in future studies.

Examination of a novel EIT variable in this study is the first step to establish EIT as a diagnostic tool to define lung disease states. Human medicine has used forced expiratory volume over maximal expiration to explain differences in global and regional ventilation before and after bronchospasmolysis (54–56). Respiratory studies in horses have used the steepness of the flow/Tidal impedance variation (F/TIV) curve and inflection point of the expiratory F/TIV curve before and after histamine provocation and salbutamol nebulization to explain differences (42). We used the peaks and troughs of the curve during inhalation and exhalation to create a ratio illustrating the differences in filling and emptying over the course of a breath. In our study we did not observe a difference in the shape of the F/TIV curves between healthy and diseased cattle during exhalation and could not gather forced expiration due to patient compliance. However, we did observe variations in the shape of the F/TIV curve during inhalation and used the quartile ventilation ratio (V_QR_) to describe those changes in the shape of the curve and filling inhomogeneities numerically.

Tidal impedance variation over one breath in another study has been shown to have a linear relationship with tidal volume in cattle (21), however, no conversion factor from impedance change to volume exists. In this study the tidal impedance change during expiration was used to define expiratory volume over time. There was no difference in TIV_EXP_ between diseased and sick cattle, therefore any differences in the fractions of ventilation relating to inhomogeneity in filling are more likely to be a true difference and not differences arising from changes in total volume or breath length.

**Figure 6:**
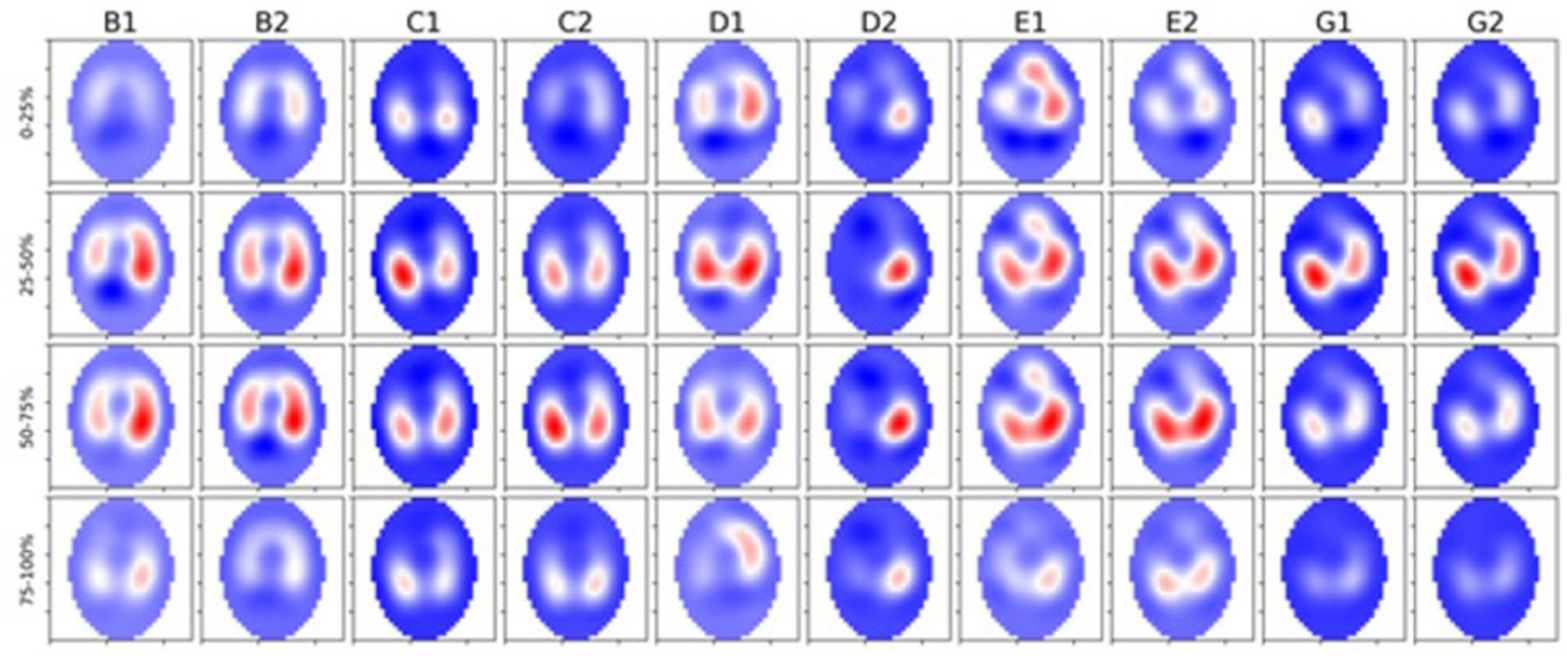
Five healthy cattle (B, C, D, E, and G) showing global images of total impedance variation over time during inhalation at 0–25%, 25–50%, 50–75%, and 75–100% of total impedance change **(top-bottom)**. Two breaths are displayed from each animal (1 and 2 preceded by the letter of each animal). The color gradient illustrates areas of no impedance change (dark blue) scaled up to areas of maximal impedance change (red).

**Figure 7:**
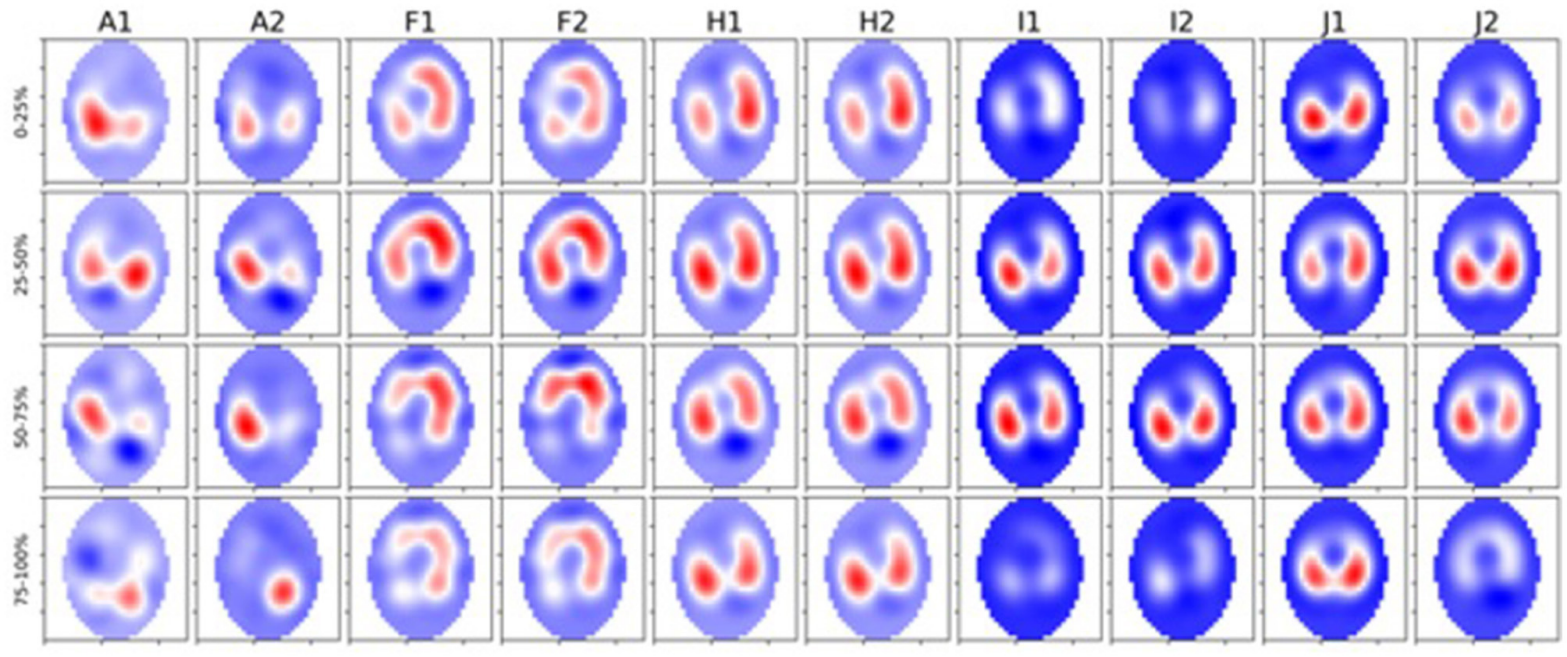
Five diseased cattle (A, F, H, I, and J) showing global images of total impedance variation over time during inhalation at 0–25%, 25–50%, 50–75%, and 75–100% of total impedance change **(top-bottom)**. Two breaths are displayed from each animal (1 and 2 preceded by the letter of each animal). The color gradient illustrates areas of no impedance change (dark blue) scaled up to areas of maximal impedance (red).

We observed a significant difference in filling time when comparing the V_QRi_ during inhalation. Healthy cattle have a lower V_QRi_ of filling when compared to diseased cattle, suggesting that in a healthy lung filling takes place over a shorter portion of the breath compared to diseased cattle. The shortened filling time is visible at 0–25% and 75–100% of the filling where minimal impedance change was measured (Figure 6). This suggests that in healthy cattle most of the filling of the lungs happens within the second and third quartile of the breath. In diseased cattle, impedance changes were observed in each quartile of the whole breath. There was no significant difference seen in the V_QRe_ (Figure 7); this may be due to the passive nature of exhalation compared to active inhalation, leading to less change. However, we would expect changes in exhalation dynamics due to airway resistance and air trapping from obstructive disease. To fully investigate this further, a larger cohort of animals would be required and further investigation into the inhomogeneity of breathing and rate of change of impedance globally and regionally over time.

With obstructive diseases such as asthma in horses the work of breathing increases due to overinflation of the lung, reduction in the expired volume, and higher inspiratory filling pressures (57, 58), leading to extended inhalation times to reach adequate filling. In our results, we observed the majority of ventilatory changes occurring during the middle portion of the breath in healthy cattle. In comparison, diseased cattle had ventilatory changes throughout the whole breath, which suggests a similar increased work of breathing where pathology may be present. Airway diameter may also be a contributing factor to ventilation changes and filling times. Cattle have increased resistance in the nares and trachea, evident in diseases such as infectious bovine rhinotracheitis, where inflammation narrows the trachea; this may further contribute to increased inhalation time and flow due to the increased resistance in the upper airway (52, 59, 60).

The shape of the inhalation F/TIV curve appeared subjectively smoother in healthy cattle with less observable peaks, which is in agreement with Lekeux, who described inhalation flow/volume curves, using mask-Fleisch pneumotachography, as more rounded (61). The flow/volume curves were derived from maximal inhalation and exhalation plotted against volume. Lekeux noted more changes in the expiratory curve in diseased cattle, which differs from the findings in our study where changes were associated with inhalation (61). The difference noted in our results may be explained by the position of measurement. EIT measures at the level of the thorax, making changes in impedance as a surrogate for volume potentially easier to detect than a pneumotachograph connected to a breathing mask measuring at the nares. The significance of these differences in the shape of the F/TIV curve are not fully understood. Initial observations do indicate a difference between cattle and horses, which contrasts from previous work. To fully understand the mechanics of breathing in cattle, we will perform further analyses on a larger cohort of animals.

### 4.1 Limitations

A limitation of this study was the small sample size, but we note that significant differences were seen in both the healthy and diseased group when comparing clinical respiratory scores and specific EIT variables. In the EIT analysis, one animal appeared to be an outlier for the V_QRi_, while in in the F/TIV curves one healthy and one diseased animal appeared to be outliers. Data from a larger cohort of animals would improve the robustness of the analysis, and comparison of EIT to other diagnostics including histology, ultrasound, and post-mortem would allow more accurate confirmation of findings.

Comparison to clinical examination is limited, as 35–50% of respiratory disease is not detected via clinical examination (8, 62); therefore the sensitivity of detection is reduced. The scoring system structured the clinical examination however, this was not a validated system and therefore the health status determination is limited from this approach. Further testing including blood testing and post-mortem analysis would improve the robustness of the system, however, this was beyond the scope of the study.

In our study, global images were examined using all pixels in the obtained image including those outside of the lungs. To provide a more accurate analysis of pixels derived from lung tissue and not heart or ruminal gas will require further delineation of regions of interest. In initial studies, creation of a global measure negates the issue of using regions of interest, however, future studies would benefit from delineation to allow more accurate analysis. In addition, regional areas and individual pixels have not been examined in this study, so there are potential regional changes which may influence the overall categorization of disease. With a single plain EIT, a lens-shaped ellipse of data is gathered and reconstructed and the position of the 5th intercostal space is used to allow the maximal area of lung tissue to be gathered, however the cranial and caudal tips of the lung will not be included in the data collected, therefore these areas will not be represented in the analysis. In future studies, development of a 3-D belt will improve data capture of the entire lung region, and improved definition of the region of interest, individual pixel analysis, and investigation of the slope of the F/TIV curve may provide greater detail to define disease.

## 5 Conclusions

Electrical impedance tomography is able to detect differences in the inhalation portion of the breath when comparing healthy and diseased cattle defined via clinical examination. These initial findings highlight the potential of EIT as a useful imaging tool for the diagnosis of respiratory disease in cattle. Future studies should involve larger cohorts and compare to other diagnostic imaging methods as well as post-mortem samples when available to confirm EIT findings.

## Data availability statement

The raw data supporting the conclusions of this article will be made available by the authors, without undue reservation.

## Ethics statement

The animal studies were approved by Murdoch University Animal Ethics Committee R3275/20. The studies were conducted in accordance with the local legislation and institutional requirements. Written informed consent was obtained from the owners for the participation of their animals in this study.

## Author contributions

OB: Data curation, Formal analysis, Investigation, Methodology, Writing—original draft, Writing—review & editing. YK: Formal analysis, Software, Writing—review & editing. AG: Formal analysis, Software, Writing—review & editing. WD: Formal analysis, Writing—review & editing. HH: Formal analysis, Software, Writing—review & editing. AR: Supervision, Writing—review & editing. MM: Conceptualization, Investigation, Methodology, Project administration, Supervision, Writing—review & editing.
